# Frequency, financial impact, and factors associated with cost outliers in intensive care units: a cohort study in Belgium

**DOI:** 10.62675/2965-2774.20250207

**Published:** 2025-01-15

**Authors:** Arnaud Bruyneel, Julie Van den Bulcke, Pol Leclercq, Magali Pirson

**Affiliations:** 1 Hospital Management and Nursing Research Deptment School of Public Health Université Libre de Bruxelles Bruxelles Belgium Health Economics, Hospital Management and Nursing Research Deptment, School of Public Health, Université Libre de Bruxelles - Bruxelles, Belgium.

**Keywords:** Cost and cost analysis, Health care economics and organizations, Hospital costs, Health police, Financial management, hospital, Diagnosis-related groups, Intensive care units, Belgium

## Abstract

**Objective:**

This study aimed to explore the association between high outliers and intensive care unit admissions and to identify the factors contributing to high intensive care unit costs.

**Methods:**

This retrospective cohort study used data from 17 Belgian hospitals from 2018 and 2019. The study focused on the 10 most frequently admitted diagnosis-related groups in the intensive care unit. The dataset included medical discharge summaries and cost per stay from the hospital perspective.

**Results:**

A total of 39,279 hospital stays were analyzed, 11,124 of which were intensive care unit admissions; additionally, 2,500 of these stays were high outliers. The proportion of high outliers was significantly greater in the intensive care unit group, and admission to the intensive care unit was significantly associated with high outliers in the multivariate analyses. Factors associated with high intensive care unit outliers included the medical diagnosis-related group category, patients from nursing homes, intensive care unit stay duration exceeding 4 days, and specific technical procedures (measurement of intracranial pressure, continuous hemofiltration, and mechanical ventilation).

**Conclusion:**

Admission to the intensive care unit increases the likelihood of being classified as an outlier, thus significantly impacting hospital costs. This study identified factors that can be used to predict intensive care unit outliers, which can enable adjustments to diagnosis-related group-based funding for intensive care units.

## INTRODUCTION

Despite there being a more limited number of intensive care unit (ICU) beds compared with general care units (representing approximately 10% to 30% of all hospital beds), ICU stays and costs represent a significant portion of inpatient hospital stays.^1-3^These costs can significantly vary depending on the organization of the ICUs within each country.^4^ In Belgium, the organization of ICUs is essentially mixed and encompasses both medical and surgical cases; additionally, there are no distinct levels of intensive care. ICU beds represent 4.7% of the hospital beds and account for a direct cost of 17.4% of all hospital wards.^5,6^

An outlier (whether low or high) is a value or observation that significantly deviates from other observations of the same phenomenon, with these values being significantly different from the ‘normally’ measured values.^7^ In the context of hospital financing, the measured value that is used to identify outliers is the hospital cost; alternatively, when this information is unavailable, the length of hospital stay can be utilized.^8^ High outliers represent a considerable challenge for health care systems and hospital administrators.^9^ Even in small sample sizes, these outliers can significantly skew means and variances, thus complicating case mix-based financing and efforts to reduce hospital stay costs via rational management strategies.^10^ Therefore, the identification and assessment of the actual costs of these high outliers is imperative for hospital and ICU financing. High outliers account for approximately 5% of admissions, between 15% and 25% of hospital days and approximately 15% of hospital expenditures, depending on the study.^11-15^ Other researchers have indicated that outlier costs are more likely to occur when certain patient factors are present. These factors include comorbidities, advanced age, living a long distance from the hospital, the presence of complications, readmission, emergency hospitalization, greater severity within the diagnosis-related group (DRG), and in-hospital mortality.^16^ Some studies have also highlighted the impact of hospital size and type on cost variations within DRGs.^17^ As an aspect of the financing of hospitals in Belgium, high outliers are identified based on a statistical threshold (Q3+2×(Q3−Q1)) that is derived from the average length of stay per DRG and severity of illness (SOI). High outliers have not been investigated as well in the ICU literature; however, given their importance for health policy and hospital management, specific research on this subject is essential.^14^

Given the considerable expenses associated with ICUs, the financing of these units plays a crucial role in health care organization and the quality of care.^18^ In Europe, there is heterogeneity in the financing mechanisms of ICUs due to their different organizational structures across various countries.^6^ A majority of countries solely utilize the DRG system as the financing mechanism for ICUs. Other countries also use DRG-based financing but with an additional payment supplement for ICU admissions. Finally, some countries exclude ICUs from DRG-based financing entirely.^19^ These three financing mechanisms may experience underfunding issues, with the first mechanism being particularly sensitive to this problem.^20^Underfunding is notably prevalent in highly specialized intensive care services, such as pediatric services, as well as in specific categories of adult patients.^19^ These financing mechanisms do not adequately consider significant outliers in the ICU. The prevalence and financial impact of these factors on hospital costs should be further investigated to refine the financing mechanisms for the ICU.

In Belgium, hospital funding combines a budget of financial means linked to hospital activity (which is evaluated by the DRG) and funding from medical procedures and pharmaceutical products.^21^ Importantly, intensive care funding in Belgium is complex and not solely based on actual ICU capacity. Financed beds with intensive care characteristics are calculated based on various factors, including extended medical services (20%), the nursing profile of intensive care (40%), and the national percentage of intensive care per all patients in refined diagnosis-related groups (APR-DRG) (40%). Consequently, a hospital may have more or fewer actual ICU beds than funded beds. The political intention in Belgium is to reform this funding model by integrating operational costs (such as nursing staff, laboratories, medical imaging, and drugs) into DRGs, thus placing importance on the financing method for ICUs.

The objectives of this study were to determine the associations between high outliers and ICU admissions and to identify the factors associated with these outliers.

## METHODS

### Study design and setting

This was a retrospective cohort study using data from 17 Belgian hospitals from January 1, 2018 to December 31, 2019. Of these 17 hospitals, 3 were teaching hospitals, and 3 were public hospitals. The number of ward beds ranged from 100 to –973, thus totaling 7,229 beds, and the number of ICU beds ranged from 9 to –39, thus totaling 332 beds. These 17 hospitals represent 18.8% of the hospital stays in Belgium. The Strengthening Reporting of Observational Studies in Epidemiology (STROBE) guidelines for cohort studies were applied.^22^

### Participants

The study included the 10 DRGs that were most frequently admitted to the ICU, with each DRG associated with 4 SOI categories, which accounted for approximately 30% of all of the ICU admissions across the 17 participating hospitals.

For the selection of outlier costs based on the total cost of the stay, high outlier costs were calculated by using the DRG and SOI (independent of ICU admission) via the following formula: 75 th percentile +1.5x the interquartile range. .^10,23^ Via the same formula (  (25th percentile −1.5x interquartile range  ), low-cost outliers were also calculated and removed from the database to avoid affecting inlier costs and to preserve cost homogeneity.

A total of 2,146 records of incomplete stays (no administrative data being observed in the hospital, very short stays < 6 hours, no available data on costs per pathology and remaining in the hospital on December 31, 2019) and 2,343 records of low outliers (5.4%) (representing 649 ICU stays) were excluded. In total, 39,279 stays were analyzed, including 2,500 high outliers (6.4%), 11,124 of which were ICU admissions (26.9%). For the homogeneity analyses, to ensure satisfactory representativeness, DRGs and SOIs with fewer than 30 stays were excluded from the analysis, thus allowing for the analysis of 9 DRGs with one to two levels of SOI and resulting in a total of 16 groups being analyzed.^23^

### Variables and data sources

The dataset included inpatient information that was extracted from medical discharge summaries, along with data on length of stay (LOS) and cost per stay, which were data collected from the hospital’s perspective. The cost from the hospital’s perspective is determined via a full costing approach using cost accounting analytical methodology.^21,24^ The full costing approach included all cost-generating activities and the resources that were needed to oversee and coordinate the department (such as doctors, nursing staff, pharmaceutical products, consumables, depreciation of equipment, radiological resources, and biological resources, among other factors). Each expense was allocated per patient via a specific key (e.g., the cost of nursing was allocated based on the length of stay and the time devoted to patient care). All of the costs in the study are expressed in euros (€). The costs in 2018 were adjusted to reflect inflation in Belgium in 2019 (2.05%).

The DRGs were obtained via 3 M grouper, which uses the APR-DRG system (version 28.0) comprising 355 different classes; moreover, it is identical across all Belgian hospitals.^25^ The SOI is determined based on the DRG complication hierarchy, which classifies the extent of organ system loss of function or physiological decompensation on a scale of minor (1), moderate (2), major (3), or extreme (4). Patient assignment to these subgroups considers not only specific secondary diagnoses but also interactions between secondary diagnoses, age, principal diagnosis, and procedures.^26^ The SOI determination is disease-specific, with high severity of illness primarily resulting from interactions between multiple conditions. The risk of mortality indicates the likelihood of death. Furthermore, a patient may exhibit severe illness but only a minor risk of mortality.

Two indicators, including the coefficient of variability (standard deviation/mean) and the coefficient of quartile variation (CQV)(Q3−Q1/Q3+Q1) , were used to measure the homogeneity of costs per stay for each DRG and each level of severity.

The primary diagnosis, which was coded with the International Classification of Diseases 10^th^ edition (ICD-10), and sociodemographic data (such as age, sex, and mortality) were extracted from the minimum hospital discharge summary and administrative data, which are identical for Belgian hospitals.^27^ Additionally, the Charlson score was computed via the same data system, which utilized ICD-10 codes and administrative data.^28^ Initially developed in 1984, the Charlson index was revisited in 2011, with authors recalibrating the weights for each comorbidity based on mortality rates that were observed in the year following hospital discharge. These updated weights were then applied to hospital discharge data from six countries, thus demonstrating promising predictive capabilities for hospital mortality.^29^ Notably, the Charlson score exhibited strong predictive accuracy for 30-day and 1-year mortality in ICU patients.^30,31^ Data on medical procedures (such as mechanical ventilation, extracorporeal membrane oxygenation (ECMO), and continuous hemofiltration) were obtained from billing files. The nomenclature of these bills is identical in Belgium and includes a unique code for each specific medical service within a specific area, including the ICU.^32^ To assess the level of social determinants of health, a scale was used based on a recently published study of secondary diagnoses in medical discharge summaries with ICD-10 codes.^33^

Stays that involved hospitalization in the ICU (regardless of duration) were referred to as “ICU patients”, and outliers among these patients were termed “ICU outliers”.

### Statistical methods

Statistical analyses were conducted via STATA® version 18 and R software version 4.1.2 (R Core Team). A p value < 0.05 was considered to be statistically significant. Descriptive statistics are presented as proportions for categorical variables, means with standard deviations (SDs) for continuous variables, and medians with interquartile ranges (IQRs) for nonnormally distributed variables. Mann-Whitney tests were used for asymmetric variables, whereas Student’s t tests and chi-square tests were employed for normally distributed variables and proportions. The Kolmogorov-Smirnov test was used to assess the symmetry of the continuous variables.

Univariate logistic regression models were used to explore associations between independent variables (sociodemographic data such as sex, emergency department admission, and nursing home residency; minimum hospital discharge summaries including the Charlson score, presence of social determinants of health, mortality risk classification of DRG, and DRG category; administrative data such as ICU LOS category and patients dying in the ICU; and accompanying data such as the measurement of intracranial pressure, continuous hemofiltration, mechanical ventilation, and its duration) and dependent variables (high outliers). All of the variables from the univariate analyses were included in the multiple logistic regression models. The area under the receiver operating characteristic curve (AUC) was calculated for the logistic regression model.

### Ethical considerations

The inpatient records that were used in the retrospective study were fully anonymized by the hospitals, and the research team did not have any access to medical files. The study was approved by the Ethics Committee of the Erasmus Hospital in Brussels, Belgium (reference: N2023/047) and was conducted according to the Helsinki Declaration and General Data Protection Regulations (EU 2016/679). Signed informed consent was obtained from all of the individual participants who were included in this study.

## RESULTS

The mean age (± standard deviation) was significantly lower for patients admitted to the ICU (65.0 ± 16.7 *versus* 70.7 ± 15.7; p value < 0.0001). The proportion of geriatric patients in the ICU patient group was significantly lower (30.1% *versus* 43.3%; p value < 0.0001), as was the proportion of nursing home residents (2.9% *versus* 8.4%; p value < 0.0001). The percentage of patients arriving via the emergency department was lower in the ICU group (49.8% *versus* 70.4%; p value < 0.0001) and in the medical category of the DRG (26.3% *versus* 71.1%; p value < 0.0001). Social determinants of health were less prevalent among ICU patients (18.3% *versus* 24.4%; p value < 0.0001), with the most frequent economic domain observed for both groups (Table 1). The Charlson scores were relatively similar, although there was a significant difference observed between the two groups. The median length of hospital stay [p25 - p75] was significantly longer in the ICU group (9.1 [5.1 - 15.1] *versus* 7.3 [4.3 - 12.1] days; p value < 0.0001). In terms of mortality risk according to the DRG classification, 45.9% of ICU patients were in the major and extreme groups, whereas 37.5% were in the non-ICU group (p value < 0.0001); additionally, the in-hospital mortality rate was also higher for ICU-admitted patients (10.0 *versus* 5.3%; p value < 0.0001). For the ICU group, the ICU mortality rate was 7.9%, with a median length of stay of 2.0 days [1.0 - 4.0]. The proportions of patients with intracranial pressure monitoring, continuous hemofiltration, and ventilation were 2.1%, 3.5%, and 52.6%, respectively (Table 1).

**Table 1:** Clinical description of the sample

Characteristics	Non-ICU patients (n = 28,155)	ICU patients (n = 11,124)	p value	Total (n = 39,279)
Men	15,119 (53.7)	6,541 (58.8)	< 0.0001	21,660 (55.1)
Age (years)	70.7 ± 15.7	65.0 ± 16.7	< 0.0001	69.1 ± 16.2
Pediatric cases (< 15 years)	282 (1.0)	234 (2.1)	< 0.0001	516 (1.3)
Geriatric cases (> 75 years)	12,191 (43.3)	3,348 (30.1)	< 0.0001	15,539 (39.6)
Patients from a nursing home	2,365 (8.4)	323 (2.9)	< 0.0001	2,688 (6.8)
Emergency department admission	19,821 (70.4)	5,540 (49.8)	< 0.0001	25,361 (64,6)
Category of DRG				
Medical	20,008 (71.1)	2,929 (26.3)	< 0.0001	22,937 (58.4)
Surgical	8,147 (28.9)	8,195 (73.7)		16,342 (41.6)
Presence of social determinant of health	6,870 (24.4)	2,036 (18.3)	< 0.0001	8,906 (22.7)
Charlson score	4.0 [3.0 - 7.0]	4.0 [2.0 - 6.0]	0.0001	4.0 [2.0-6.0]
Hospital LOS (days)	7.3 [4.3 - 12.1]	9.1 [5.1 - 15.1]	< 0.0001	7.9 [4.4 - 13.0]
Risk mortality classification of DRG				
Minor or moderate	17,597 (62.5)	6,018 (54.1)	< 0.0001	23,615 (60.1)
Major or extreme	10,558 (37.5)	5,106 (45.9)		15,664 (39.7)
Hospital mortality	1,492 (5.3)	1,112 (10.0)	< 0.0001	2,605 (6.6)
ICU characteristics				
ICU mortality	-	883 (7.9)	-	-
ICU LOS (days)	-	2.0 [1.0 - 4.0]	-	-
Measurement of intracranial pressure	-	234 (2.1)	-	-
ECMO	-	4 (-)	-	-
Continuous hemofiltration	-	388 (3.5)	-	-
Ventilated patients	-	5,847 (52.6)	-	-
Mechanical ventilation time (days)	-	2.0 [1.0 - 3.0]	-	-

ICU - intensive care unit; DRG - diagnosis-related group; LOS - length of stay; ECMO - extracorporeal membrane oxygenation. The results are expressed as n (%), mean ± standard deviation and median [p25 - p75].

DRGs associated with neurosurgery (021 and 024) and cardiac surgery (163 and 166) had frequent ICU admissions in this study. However, the DRG with the highest prevalence of high outliers was a medical DRG known as the chronic obstructive pulmonary disease DRG (140) in SOI 3, wherein 13.1% of cases were identified as being high outliers. Almost half of these patients were admitted to the ICU, which constituted 11.0% of the ICU outlier sample (Table 1S - Supplementary Material).

**Figure 1:**
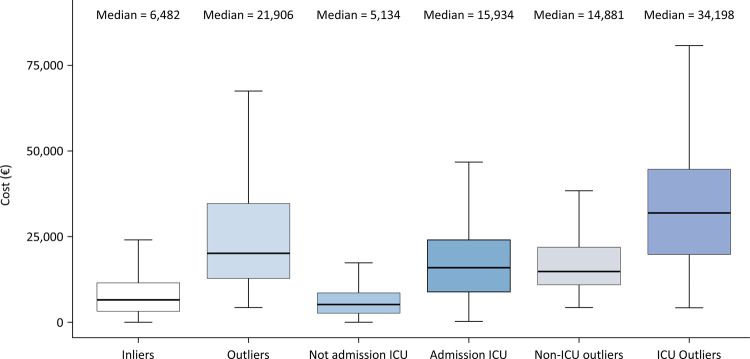
Comparison of total cost of stay according to the type of admission (intensive care unit or nonintensive care unit) and to the type of stay (outliers or inliers).

**Figure 2:**
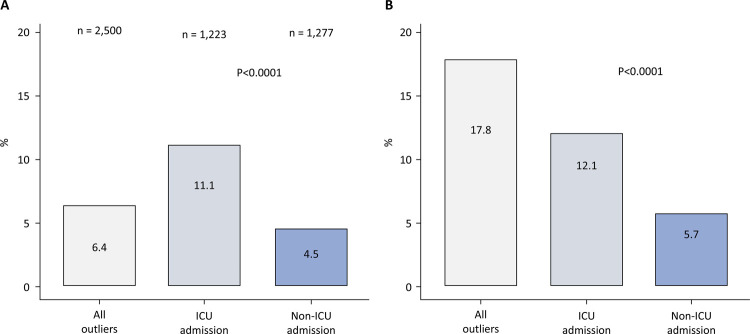
Proportion of high outliers according to the type of admission (intensive care unit or nonintensive care unit) (A) and proportion of the cost of high outliers according to the type of admission (intensive care unit or nonintensive care unit) relative to the total cost for all admissions (B).

The median cost per stay for the outliers was €21,906 [€13,140 - €37,473] , and it was €6,5482 [€3,453 - €11,754] for inliers. For ICU outliers *versus* non-ICU outliers, the median cost per stay was €34,198 [€21,214 - €49,122] versus €14,881 [€10,967 - €22,346] (Figure 1). The proportion of high outliers was significantly greater in the ICU group than in the non-ICU group (11.0% *versus* 4.5%, respectively; p value < 0.0001). The proportion of the cost of high outliers represented 12.1% of the total cost for ICU patients and 6.0% of the total cost for non-ICU patients (Figure 2).

ICU - intensive care unit.

ICU - intensive care unit.

Analysis of length of stay and costs according to DRG and SOI revealed that the DRG “cardiac valve procedures without cardiac catheterization (163)” (SOI 4) demonstrated the highest median cost per stay for the two groups. With respect to the length of stay, the DRGs “major stomach, esophageal & duodenal procedures (221)” (SOI 4), “craniotomy except for trauma (021)” (SOI 4), and “coronary bypass without cardiac catheter or percutaneous cardiac procedure (166)” (SOI 4) demonstrated the highest LOS for all of the samples (Table 2S - Supplementary Material).

In the multivariate analyses, admission to the ICU was significantly associated with high outliers (OR = 3.43, 95%CI 3.06 - 3.85) , as well as), the medical DRG category (OR = 3.83, 95%CI 3.35 - 4.37) , length of stay (OR = 1.11, 95%CI 1.10 - 1.12) , and deceased patients (OR = 1.76, 95%CI 1.49-2.10) (Table 3S - Supplementary Material). In terms of factors associated with high ICU outliers, the medical DRG category (OR = 1.93, 95%CI 1.48 - 2.51) , patients from nursing homes (OR = 1.96, 95%CI 1.53 - 2.46) , an ICU stay duration exceeding 4 days (OR = 3.16, 95%CI 2.32 - 4.31) , and specific technical procedures in the ICU (the measurement of intracranial pressure, continuous hemofiltration, and duration of mechanical ventilation) were significantly associated with high ICU outliers. With respect to the probability of predicting ICU outliers, the area under the ROC curve for the multivariate model was 0.82 (95%CI: 0.80 - 0.84) (Table 2).

**Table 2:** Factors associated with high outliers for intensive care unit admission

Variables	OR nonadjusted (95%CI)	OR adjusted (95%CI)
Charlson score		
0	Reference	Reference
0 - 3	1.22 (0.94 - 1.59)	1.18 (0.78 - 1.79)
4 - 5	1.27 (0.97 - 1.68)	0.84 (0.54-1.29)
6+	1.65 (1.26 - 2.15)†	1.09 (0.71 - 1.68)
Presence of social determinant of health	1.74 (1.51 - 1.99)‡	1.22 (0.98 - 1.52)
Mortality risk classification of DRG		
Minor or moderate	Reference	Reference
Major or extreme	1.74 (1.54 - 1.96)‡	0.38 (0.29 - 0.50)‡
DRG category		
Surgical	Reference	Reference
Medical	1.85 (1.63 - 2.08)‡	1.93 (1.48 - 2.51)‡
Gender		
Female	Reference	Reference
Male	0.79 (0.70 - 0.89)‡	0.75 (0.62 - 0.90)‡
Emergency department admission	2.01 (1.78 - 2.27)‡	1.96 (1.53 - 2.46)‡
From a nursing home	1.36 (0.99 - 1.87)	-
ICU LOS (days) category		
≤ 1	Reference	Reference
2	0.78 (0.62 - 1.23)	0.99 (0.70 - 1.39)
3	0.95 (0.73 - 1.22)	0.71 (0.47 - 1.06)
4	1.45 (1.11 - 1.89)	1.04 (0.66 - 1.62)
> 4	5.29 (4.52 - 6.17)‡	3.16 (2.32 - 4.31)‡
Patients dying in the ICU	1.67 (1.38 - 2.03)‡	0.63 (0.47-0.83)‡
Patents with measurements of intracranial pressure	1.85 (1.31 - 2.60)‡	1.27 (1.12 - 1.42)*
Patents with continuous hemofiltration	5.03 (4.04 - 6.27)‡	2.18 (1.59 - 2.98)‡
Patients on mechanical ventilation	1.82 (1.62 - 2.06)‡	-
Mechanical ventilation time LOS (days) category		
1	Reference	Reference
2	1.94 (1.49 - 2.53)‡	1.81 (1.37 - 2.40)‡
3	2.58 (1.74 - 3.83)‡	1.64 (1.06 - 2.52)‡
4	3.11 (2.27 - 4.26)‡	2.72 (1.94 - 3.82)‡
> 4	10.50 (8.23 -1 3.38)‡	5.19 (3.85 - 7.00)‡
Area under ROC curve for model multivariate (95%CI)		0.82 (0.80 - 0.84)

OR - odds ratio; ICU - intensive care unit; DRG - diagnosis-related group; LOS - length of stay. * p value < 0.05; † p value < 0.01; ‡ p value < 0.001.

**Figure 3:**
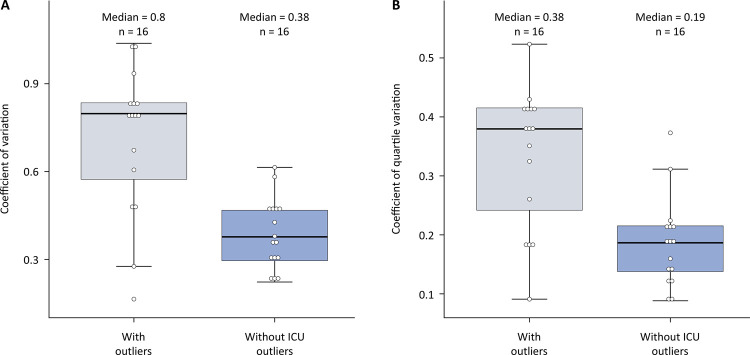
Variation in cost according to the diagnosis-related group and severity of illness with and without intensive care unit outliers for the coefficient of variation (A) and the coefficient of quartile variation (B).

With respect to the analysis of cost homogeneity according to DRG and SOI, the coefficient of variation (CV) improved significantly with the removal of ICU outliers (0.80 [0.56 - 0.90] versus 0.38 [0.300.47]) . Similarly, the coefficient of quartile variation (CQV) was also better with the omission of high outliers in the ICU than without outliers (0.38 [0.310.42] versus 0.19 [0.140.22]) (Figure 3).

After exclusion of diagnosis-related groups and severity of illness levels without at least 30 stays.

ICU - intensive care unit.

## DISCUSSION

The objective of this study was to assess the impact of ICU admission on high outliers and identify the factors associated with these outliers. For the main results of the study, the cost per stay for ICU outliers was more than 2X that for non-ICU outliers. High outliers were more common in the ICU group (11.0%) than in the non-ICU group (4.5%), whereby they accounted for 12.1% and 6.0% of the total costs, respectively. Multivariate analyses revealed a strong association between ICU admission and high outliers. ICU admission, longer ICU stays, nursing home residencies, and specific ICU procedures were significantly associated with high rates of ICU outliers. The exclusion of high ICU outliers improved cost homogeneity in the analysis.

The ICU sociodemographic and medical severity data (including age, Charlson score, mortality rate, proportion of patients on mechanical ventilation, and duration of ventilation) were very similar to data from other studies conducted in Belgian ICUs without specific inclusion criteria, as occurred in this study.^34,35^ However, compared with other European countries, the rates of mechanical ventilation, mortality, and length of stay were lower, thus suggesting a lower degree of severity in our ICU sample (ICON and SOAP investigators et al., 2018).^36^ There were significant differences observed in sociodemographic data between patients who were admitted to the ICU and those who were not admitted. The mean age, proportion of nursing home patients, proportion of emergency department patients, and proportion of social determinants of health were greater for non-ICU patients, thus implying a certain selection of patients for ICU admission, even in the absence of official admission criteria in the country. These differences between ICU and non-ICU patients can also be explained by a greater prevalence of surgical categories in the ICU, such as DRGs for neurosurgery (021 and 024) and cardiac surgery, which most likely resulted from the selection of the 10 most frequently admitted DRGs to the ICU (163, 166).

The prevalence of high outliers, which was 6.4% for our entire sample, closely resembled findings from other studies on this topic.^9,37^ We observed 2X the proportion of high outliers in the ICU, which is a significant increase compared with other studies that have been performed in this unit.^14^ As shown by figure 2, high outliers have a substantial effect on hospital costs, with a cost of approximately €50 million being solely observed for high ICU outliers, which represents more than 12% of the cost per stay for the entire sample. When the median cost per patient was examined, the outlier values in the ICU were twice as high as the non-ICU outlier values and were up to five times higher than the inliers for all of the samples. The cost differences between the categories of outliers do not seem to be explained by a greater median length of stay (IQR) in the hospital when comparing high-ICU outliers and non-ICU outliers (25.6 [10.9 - 37.7] versus 24.9 [14.7 - 39.0] , respectively); rather, the cost differences may be due to differences in the procedures that were performed between these two groups, such as surgery and specific ICU procedures (including continuous hemofiltration and mechanical ventilation), which increase the cost per patient.^13,38,39^ Our approach to selecting outliers yielded higher proportions of cases than other studies. In these reports, outlier rates typically range between 2% and 8%, and they depend on factors such as the country, the utilized DRG version, the year of study, and the method that was employed for outlier selection.^16^ Another study reported rates ranging from 2.06% to 5.9%. These rates can vary depending on the different statistical methods that are used for outlier identification.^40^

Several factors associated with high outliers were analyzed in this study. First, for all of the high outliers, admission to the ICU resulted in a 3.5-fold greater risk according to multivariate analyses. This result confirms the significant impact of ICU admission on high outlier status.^9,10^ Deceased patients, length of hospital stay, and medical category of the DRG were also significantly associated with high outliers. With respect to factors that were specifically associated with high outliers for ICU admissions, admissions from the emergency department, a length of stay exceeding 4 days in the ICU, continuous hemofiltration, intracranial pressure measurement, and mechanical ventilation were deemed to be significant in our multivariate model. The combination of factors resulted in a satisfactory prediction of ICU outliers, with an AUC greater than 0.80.

The identification of cost outliers is almost impossible when patients are hospitalized because the costs per stay are not yet available.

These clinical factors should make it possible to identify outliers for which the costs are not available and could represent exclusion criteria for global funding per disease, thus potentially justifying specific funding to cover the additional costs of these outliers. Our results also provide a methodology for estimating ICU inpatient stays, which should be subject to specific measurements in terms of funding and medical management to reduce their impact on ICU costs (if possible).

Cost homogeneity analyses according to the DRG and SOI were conducted to understand the impact of outliers on these measures. Regardless of whether the coefficient of variation or the coefficient of quartile variation was used, the removal of outliers led to a significant decrease in these values. When regarding the coefficient of variation, although the threshold is still controversial for the SOI, we utilized a threshold of 0.30, which is often used in the literature to assess good homogeneity.^23,41^

Finally, these results confirm a significant association between admission to the ICU and high outliers. Approximately 1 in 10 ICU admissions was indicated as a high outlier, with a consequent impact on hospital costs and cost homogeneity being observed according to the DRG and SOI. Therefore, it is essential to identify and isolate these cases by using the identified factors in this study, given that the costs per stay are not immediately known. Although further research is needed to confirm the predictive accuracy of these factors, our current results show that DRG-based funding for ICUs in Belgium is feasible. However, funding for high outliers should be additionally provided, and their identification should be performed during the hospital stays of patients based on clinical criteria.

### Limitations and strengths

This study has certain limitations. First, the variables that were included in the model were retrospectively derived from hospital databases, rather than being prospectively collected. Patient variables were obtained from billing data and may not always have accurately reflected the actual services provided to the patient because of the invoicing rules (such as nonaccumulation of billing information for certain medical procedures). Second, caution is advised when comparing cost calculations with other published studies, as the utilized methodology for calculating costs and the chosen perspective may vary. Third, anonymous administrative data lack information on patient medical severity, which is important for predicting hospital costs and risks. However, our multivariate model included data on pathology, ICU-specific treatments, hemofiltration, and comorbidities. Fourth, the factors that are associated with high outliers were not tested with a proportional score by adapting to the forces of association; therefore, the predictivity of the identified factors should be approached with caution. Fifth, as a general rule, when a payment model is based on APR-DRG, it only applies to inliers, and it also provides additional payments for outliers. This aspect was not considered in this study and merits further analysis. Finally, caution should be exercised when interpreting the results, as impressions are often gleaned from hospital accounting data.

## CONCLUSION

A strong association was identified between intensive care unit admission and the observation of high-cost outliers in a multivariate model. Clinical factors were identified to isolate high outliers with relatively good predictivity. With additional funding devoted for high outliers, it appears that the funding of intensive care units based on diagnosis-related groups is feasible in Belgium.

## References

[1] Tan SS, Bakker J, Hoogendoorn ME, Kapila A, Martin J, Pezzi A (2012). Direct cost analysis of intensive care unit stay in four european countries: applying a standardized costing methodology. Value Health.

[2] Mastrogianni M, Galanis P, Kaitelidou D, Konstantinou E, Fildissis G, Katsoulas T (2021). Factors affecting adult intensive care units costs by using the bottom-up and top-down costing methodology in OECD countries: a systematic review. Intensive Crit Care Nurs.

[3] Kiliç M, Yüzkat N, Soyalp C, Gülhas N (2019). Cost analysis on intensive care unit costs based on the length of stay. Turk J Anaesthesiol Reanim.

[4] Reis Miranda D, Jegers M (2012). Monitoring costs in the ICU: a search for a pertinent methodology. Acta Anaesthesiol Scand.

[5] Bruyneel A, Larcin L, Martins D, Van Den Bulcke J, Leclercq P, Pirson M (2023). Cost comparisons and factors related to cost per stay in intensive care units in Belgium. BMC Health Serv Res.

[6] Marshall JC, Bosco L, Adhikari NK, Connolly B, Diaz JV, Dorman T (2017). What is an intensive care unit? A report of the task force of the World Federation of Societies of Intensive and Critical Care Medicine. J Crit Care.

[7] Smiti A (2020). A critical overview of outlier detection methods. Comput Sci Rev.

[8] Freitas A, Silva-Costa T, Lopes F, Garcia-Lema I, Teixeira-Pinto A, Brazdil P (2012). Factors influencing hospital high length of stay outliers. BMC Health Serv Res.

[9] Pirson M, Dramaix M, Leclercq P, Jackson T (2006). Analysis of cost outliers within APR-DRGs in a Belgian general hospital: two complementary approaches. Health Policy.

[10] Pirson M, Schenker L, Martins D, Dung D, Chalé JJ, Leclercq P (2013). What can we learn from international comparisons of costs by DRG?. Eur J Health Econ.

[11] Ahmad D, Moeller K, Chowdhury J, Patel V, Yoo EJ (2018). Impact of outlier status on critical care patient outcomes: does boarding medical intensive care unit patients make a difference?. J Crit Care.

[12] Cots F, Elvira D, Castells X, Dalmau E (2000). Medicare's DRG-weights in a European environment: the Spanish experience. Health Policy.

[13] Cyganska M (2016). The impact factors on the hospital high length of stay outliers. Proc Econon Finan.

[14] Dahl D, Wojtal GG, Breslow MJ, Holl R, Huguez D, Stone D (2012). The high cost of low-acuity ICU outliers. J Healthc Manag.

[15] Marbus SD, Schweitzer VA, Groeneveld GH, Oosterheert JJ, Schneeberger PM, van der Hoek W (2020). Incidence and costs of hospitalized adult influenza patients in The Netherlands: a retrospective observational study. Eur J Health Econ.

[16] Cots F, Mercadé L, Castells X, Salvador X (2004). Relationship between hospital structural level and length of stay outliers. Implications for hospital payment systems. Health Policy.

[17] Mehra T, Müller CT, Volbracht J, Seifert B, Moos R (2015). Predictors of high profit and high deficit outliers under SwissDRG of a tertiary care center. PLoS One.

[18] Bittner MI, Donnelly M, van Zanten AR, Andersen JS, Guidet B, Trujillano Cabello J (2013). How is intensive care reimbursed? A review of eight European countries. Ann Intensive Care.

[19] Ettelt S, Ellen N (2010). Funding intensive care -- approaches in systems using diagnosis-related groups.

[20] Stephani V, Quentin V, Van den Heede K, Van de Voorde C, Geissler A (2018). Payment methods for hospital stays with a large variability in the care process.

[21] Durant G, Leclercq P, Pirson M (2021). Le financement des hôpitaux et de l'activité médicale: panorama international et principes méthodologiques.

[22] Cuschieri S (2019). The STROBE guidelines. Saudi J Anaesth.

[23] Pirson M, Delo C, Di Pierdomenico L, Laport N, Biloque V, Leclercq P (2013). Variability of nursing care by APR-DRG and by severity of illness in a sample of nine Belgian hospitals. BMC Nurs.

[24] Pirson M, Leclercq P (2014). Un projet pilote d'évaluation des coûts par pathologie, le projet PACHA. Healthcare Executive.

[25] Santos JV, Viana J, Pinto C, Souza J, Lopes F, Freitas A (2022). All Patient Refined-Diagnosis Related Groups' (APR-DRGs) Severity of Illness and Risk of Mortality as predictors of in-hospital mortality. J Med Syst.

[26] Souza J, Santos JV, Canedo VB, Betanzos A, Alves D, Freitas A (2020). Importance of coding co-morbidities for APR-DRG assignment: focus on cardiovascular and respiratory diseases. Health Inf Manag.

[27] Hirsch JA, Nicola G, McGinty G, Liu RW, Barr RM, Chittle MD (2016). ICD-10: History and Context. AJNR Am J Neuroradiol.

[28] Charlson ME, Pompei P, Ales KL, MacKenzie CR (1987). A new method of classifying prognostic comorbidity in longitudinal studies: development and validation. J Chronic Dis.

[29] Quan H, Li B, Couris CM, Fushimi K, Graham P, Hider P (2011). Updating and validating the Charlson comorbidity index and score for risk adjustment in hospital discharge abstracts using data from 6 countries. Am J Epidemiol.

[30] Christensen S, Johansen MB, Christiansen CF, Jensen R, Lemeshow S (2011). Comparison of Charlson comorbidity index with SAPS and APACHE scores for prediction of mortality following intensive care. Clin Epidemiol.

[31] Stavem K, Hoel H, Skjaker SA, Haagensen R (2017). Charlson comorbidity index derived from chart review or administrative data: agreement and prediction of mortality in intensive care patients. Clin Epidemiol.

[32] Leclercq P, Bardiaux S, Azzi D, Van Den Bulcke J, Pirson M, Chaabane S, Cousein E, Wieser P (2022). Healthcare Systems.

[33] Agarwal AR, Prichett L, Jain A, Srikumaran U (2023). Assessment of Use of ICD-9 and ICD-10 Codes for Social Determinants of Health in the US, 2011-2021. JAMA Netw Open.

[34] Bruyneel A, Larcin L, Tack J, Van Den Bulke J, Pirson M (2022). Association between nursing cost and patient outcomes in intensive care units: a retrospective cohort study of Belgian hospitals. Intensive Crit Care Nurs.

[35] Mertens K, Morales I, Catry B (2013). Infections acquired in intensive care units: results of national surveillance in Belgium, 1997-2010. J Hosp Infect.

[36] Vincent JL, Lefrant JY, Kotfis K, Nanchal R, Martin-Loeches I, Wittebole X, Sakka SG, Pickkers P, Moreno R, Sakr Y, ICON and SOAP investigators, SOAP investigators (2018). Comparison of European ICU patients in 2012 (ICON) versus 2002 (SOAP). Intensive Care Med.

[37] Pirson M, Martins D, Jackson T, Dramaix M, Leclercq P (2006). Prospective casemix-based funding, analysis and financial impact of cost outliers in all-patient refined diagnosis related groups in three Belgian general hospitals. Eur J Health Econ.

[38] Karabatsou D, Tsironi M, Tsigou E, Boutzouka E, Katsoulas T, Baltopoulos G (2016). Variable cost of ICU care, a micro-costing analysis. Intensive Crit Care Nurs.

[39] McLaughlin AM, Hardt J, Canavan JB, Donnelly MB (2009). Determining the economic cost of ICU treatment: a prospective "micro-costing" study. Intensive Care Med.

[40] Cots F, Elvira D, Castells X, Sáez M (2003). Relevance of outlier cases in case mix systems and evaluation of trimming methods. Health Care Manag Sci.

[41] Motte S, Mélot C, Di Pierdomenico L, Martins D, Leclercq P, Pirson M (2016). Predictors of costs from the hospital perspective of primary pulmonary embolism. Eur Respir J.

